# 524. Hepatic Safety of Remdesivir Across Phase 3 Placebo-controlled COVID-19 Studies

**DOI:** 10.1093/ofid/ofad500.593

**Published:** 2023-11-27

**Authors:** Lindsey Force, Chris Blair, Joshua Duckworth, Mazin Abdelghany, Ngoc-Quyen Nguyen, Robert H Hyland, Santosh Davies, Shuguang Chen, Yang Zhao, Olivia Fu

**Affiliations:** Gilead Sciences, Inc., Foster City, California; Gilead Sciences, Inc., Foster City, California; Gilead Sciences, Inc., Foster City, California; Gilead Sciences, Inc., Foster City, California; Gilead Sciences, Inc., Foster City, California; Gilead Sciences, Inc., Foster City, California; Gilead Sciences, Inc., Foster City, California; Gilead Sciences, Inc, Foster City, California; Gilead Sciences, Inc., Foster City, California; Gilead Sciences, Inc., Foster City, California

## Abstract

**Background:**

Remdesivir (RDV), an RNA polymerase inhibitor approved to treat COVID-19, is extensively metabolized by the liver. Because COVID-19 can impact hepatic function and Phase 1 RDV trials showed transient Grade 1-2 transaminase elevations, subsequent studies have aimed to assess the hepatic safety of RDV. Evaluations of the pharmacokinetics (PK) of RDV and its metabolites in healthy individuals and those with hepatic impairment have revealed no clinically relevant PK increases or new safety concerns. Postmarketing exposure based on sales data for a 5-day regimen of RDV is estimated to be >4 million patients, with no safety signals of hepatotoxicity observed in the COVID-19 population.

**Methods:**

Hepatic adverse events (AEs) and laboratory abnormalities were collected from 3 randomized, double-blind, placebo-controlled, Phase 3 clinical studies in participants ≥ 12 years of age with COVID-19 (**Table 1**). In ACTT-1, hospitalized participants (randomized 1:1) received a 10-day course of RDV or placebo. In PINETREE, participants with ≥ 1 risk factor for disease progression (randomized 1:1) received a 3-day course of RDV or placebo in an outpatient setting. In REDPINE, hospitalized participants with severe kidney impairment (randomized 2:1) received a ≤ 5-day course of RDV or placebo. In all 3 studies, RDV was given intravenously at 200 mg on Day 1 and 100 mg daily thereafter. Participants were excluded if alanine aminotransferase (ALT) or aspartate aminotransferase (AST) levels were ≥ 5 times the upper limit of normal.

**Figure:**
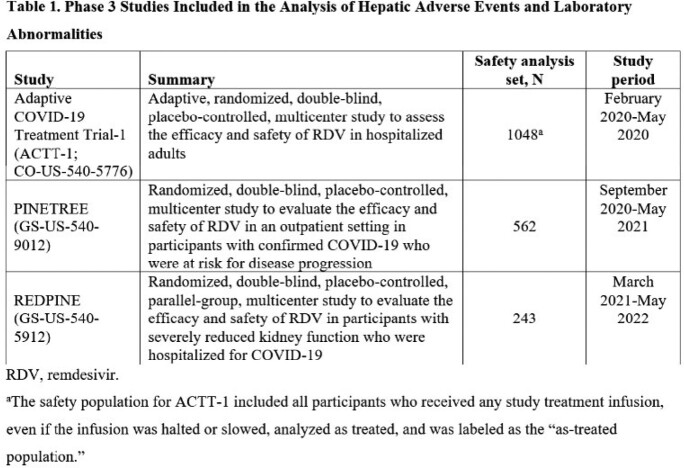

**Results:**

Hepatic AEs were reported at similar rates for each treatment group in ACTT-1 (RDV: 71 [13%]; placebo: 80 [16%]) and PINETREE (RDV: 1 [0.4%]; placebo: 4 [1.4%]; **Table 2**). In REDPINE, 12 (7.4%) and 2 (2.5%) participants in the RDV and placebo groups, respectively, reported hepatic AEs, most of which were increased levels of ALT/AST-related terms. In all 3 studies, laboratory abnormalities of increased levels of ALT, AST, and bilirubin of any grade were each reported in similar or lower percentages with RDV compared with placebo (**Table 3**).

**Figure:**
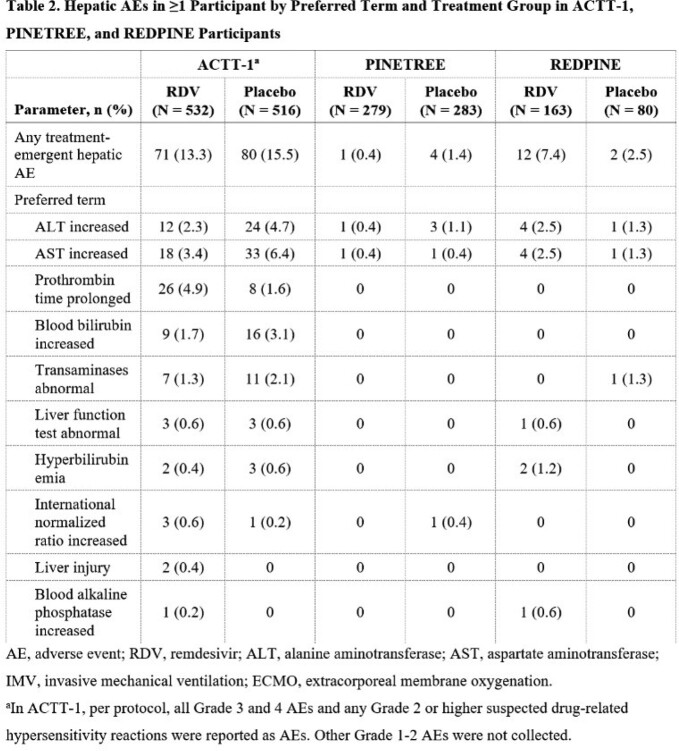

**Figure:**
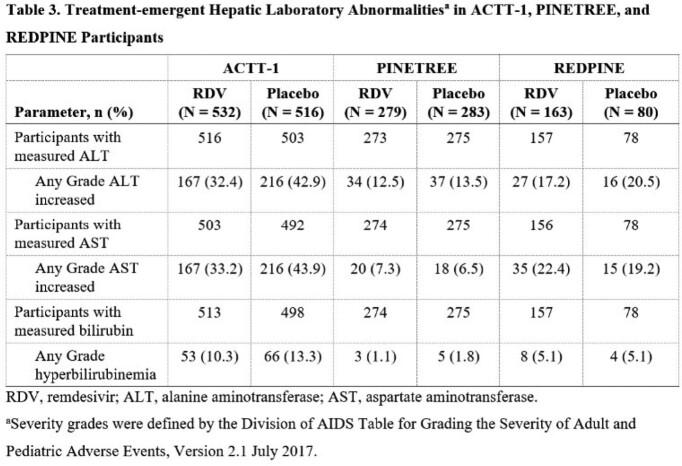

**Conclusion:**

Review of 3 placebo-controlled Phase 3 studies, as well as postmarketing safety monitoring, demonstrate the hepatic safety of RDV and characterize the background incidence of liver function test abnormalities in those with COVID-19.

**Disclosures:**

**Lindsey Force, MD**, Gilead Sciences, Inc.: Employee|Gilead Sciences, Inc.: Stocks/Bonds **Chris Blair, MS**, Gilead Sciences, Inc.: Employee|Gilead Sciences, Inc.: Stocks/Bonds **Joshua Duckworth, PhD**, Gilead Sciences, Inc.: Employee|Gilead Sciences, Inc.: Stocks/Bonds **Mazin Abdelghany, MD**, Gilead Sciences, Inc.: Employee|Gilead Sciences, Inc.: Stocks/Bonds **Ngoc-Quyen Nguyen, PharmD**, Gilead Sciences, Inc.: Employee|Gilead Sciences, Inc.: Stocks/Bonds **Robert H. Hyland, DPhil**, Gilead Sciences, Inc.: Employee|Gilead Sciences, Inc.: Stocks/Bonds **Santosh Davies, MD**, Gilead Sciences, Inc.: Employee|Gilead Sciences, Inc.: Stocks/Bonds **Shuguang Chen, PhD**, Gilead Sciences, Inc.: Employee|Gilead Sciences, Inc.: Stocks/Bonds **Yang Zhao, PhD**, Gilead Sciences, Inc.: Employee|Gilead Sciences, Inc.: Stocks/Bonds **Olivia Fu, MD**, Gilead Sciences, Inc.: Employee|Gilead Sciences, Inc.: Stocks/Bonds

